# A retrospective analysis of the effects of different analgesics on the pain of patients with traumatic thoracolumbar fractures in the peri-treatment period

**DOI:** 10.1186/s13018-021-02401-w

**Published:** 2021-04-17

**Authors:** Hao Yuan, Quan-Yuan Chang, Jie Chen, Ya-Ting Wang, Zong-Jin Gan, Song Wen, Ting-Ting Li, Liu-Lin Xiong

**Affiliations:** 1 Department of Spinal Surgery, The Affiliated Hospital of Zunyi Medical University, Zunyi, 563000 Guizhou China; 2 Department of Anesthesiology, The Affiliated Hospital of Zunyi Medical University, Zunyi, 563000 Guizhou China; 3 Institute of Neurological Disease, Department of Anesthesiology, West China Hospital, Sichuan University, Chengdu, 610044 China; 4 Department of Anesthesiology, Southwest Medical University, Luzhou, 646000 China

**Keywords:** Traumatic thoracolumbar fracture, Acetaminophen dihydrocodeine, Celecoxib, Etoricoxib, Pain degree

## Abstract

**Objective:**

To analyze and compare the effects of peri-treatment analgesics on acute and chronic pain and postoperative functional recovery of patients with thoracolumbar fractures, so as to guide the clinical drug use.

**Methods:**

Seven hundred nineteen patients with thoracolumbar fractures were collected and divided into acetaminophen dihydrocodeine, celecoxib, and etoricoxib groups. The main indicators were the degree of postoperative pain (visual analog scale (VAS)), the incidence of chronic pain and postoperative functional recovery (Oswestry dysfunction index (ODI) and Japanese Orthopedics Association score (JOA)), which were continuously tracked through long-term telephone follow-up. The correlation analysis of ODI-pain score, peri-treatment VAS score, and ODI index was performed, and bivariate regression analysis was conducted to understand the risk factors for chronic pain.

**Results:**

Regression analysis showed that severe spinal cord injury and peri-treatment use of acetaminophen dihydrocodeine were both one of the risk factors for postoperative chronic pain. But there were no statistically conspicuous differences in basic characteristics, preoperative injury, and intraoperative conditions. Compared with the other two groups, patients in the acetaminophen dihydrocodeine group had longer peri-therapeutic analgesic use, higher pain-related scores (VAS 1 day preoperatively, VAS 1 month postoperatively, and ODI-pain 1 year postoperatively), higher VAS variation, higher incidence of chronic pain 1 year after surgery, and higher ODI index. And other ODI items and JOA assessments showed no statistically significant differences. In addition, the correlation analysis showed that the peri-treatment pain score was correlated with the severity of postoperative chronic pain.

**Conclusion:**

Although the peri-treatment analgesic effect of acetaminophen dihydrocodeine is good, it is still necessary to combine analgesics with different mechanisms of action for patients with severe preoperative pain of thoracolumbar fracture, so as to inhibit the incidence of postoperative chronic pain and improve the quality of postoperative rehabilitation.

## Introduction

Traumatic thoracolumbar fracture is a serious spinal injury that can involve the middle column, resulting in spinal instability, deformity, and nerve function injury [1]. According to the survey, more than 160,000 people in North America and tens of thousands of people in China are hospitalized for traumatic thoracolumbar fractures every year [2], which is not conducive to economic development and people’s happiness. For that the effects of pain on the body, not only in the sense of discomfort, but also can lead to anxiety, limited physical activity, and other conditions. Standardized and personalized use of correct analgesics plays an important role in the rapid recovery of clinical surgery. Therefore, the choice of analgesics during the peri-treatment period is particularly important.

In fracture surgery, acetaminophen dihydrocodeine, celecoxib, and etoricoxib are commonly used in clinical analgesics. Acetaminophen dihydrocodeine, a compound preparation composed mainly of acetaminophen and dihydrocodeine tartaric acid, is widely used for all kinds of pain, especially in the acute pain caused by fractures, with poor anti-inflammatory effect [3–5]. Celecoxib and etoricoxib, both non-steroidal anti-inflammatory drugs (NSAID), are selective cycox-2 inhibitors with anti-inflammatory analgesic and antipyretic functions for traumatic fracture patients, which promote postoperative wound fast recovery, and the incidence of gastrointestinal adverse reactions caused by low [6, 7]. From the effects of the above-mentioned analgesics, it can be understood that different types of analgesics have different analgesic effects on trauma. In addition, a number of studies have shown that poor management of acute pain after surgery can lead to chronic pain in patients [8]. And compared with acute pain after surgery, chronic pain has a greater impact on postoperative psychology and quality of life [9]. However, there has been no study comparing the effects of the use of the above-mentioned three drugs on the analgesic effect and postoperative rehabilitation after thoracolumbar surgery, especially the occurrence of chronic pain. And clinically, there is no clear guideline for the use of analgesic drugs after thoracolumbar fracture surgery, including which analgesic drugs to use, how long to use, how to reduce or stop the drug, and its long-term postoperative recovery has not been reported. So, this article will focus on analyzing the effects of peri-treatment analgesics on the pain and recovery status of patients with thoracolumbar fractures after surgery, so as to provide guide clinical medication and achieve more perfect surgical recovery.

## Materials and methods

### Selection and grouping of patients

A total of 719 patients with thoracolumbar fractures from November 2009 to January 2019 were collected from the Affiliated Hospital of Zunyi Medical University in Guizhou Province. All the patients were treated for thoracolumbar fractures by the same medical group under general anesthesia and underwent posterior unilateral laminectomy with small fenestration and intrathoracic bone grafting, under the help of a minimally invasive high-definition microscope (this surgical method has been incorporated into the new technology of the Affiliated Hospital of Zunyi Medical University). The specific surgical methods include posterior pedicle screw distraction and reduction, unilateral spinal cord nerve decompression, and vertebral bone grafting and shaping. All patients were given the same discharge instructions after surgery ((1) assisted hyperbaric oxygen to promote the recovery of spinal nerve function; (2) reasonable diet and balanced nutrition; (3) keep a regular schedule and avoid tiredness; (4) regular and reasonable rehabilitation training). This study was approved by the Ethics Committee of the Affiliated Hospital of Zunyi Medical College (2015 Review No. 23). According to the analgesic drugs used during the perioperative period, the patients were divided into three groups: the acetaminophen dihydrocodeine group (the codeine group is hereafter abbreviated, *n* = 476), the celecoxib group (*n* = 130), and the etoricoxib group (*n* = 113).

#### Inclusion criteria

(1) Patients with thoracolumbar fracture Tlics score > 4 (Table 1) [10], under general anesthesia, the posterior unilateral lamina interlamellar space with small fenestration was performed for vertebroplasty in vivo; (2) the patients ranged in age from 18 to 80 years; (3) preoperative American Society of Anesthesiologists grade II–III.

**Table 1:** Tlics standard scale

Type		Point
Injury morphology	Compression	1
	Burst	2
	Translation/rotation	3
	Distraction	4
Posterior ligamentous complex	Intact	0
	Suspected/indeterminate	2
	Injured	3
Neurologic status	Intact	0
	Nerve root	2
	Cord, conus medullaris, complete	2
	Cord, conus medullaris, incomplete	3
	Cauda equine	3
Treatment options (total score)	Non-operative treatment	≤ 3
	Non-operative or surgery	4
	Surgical intervention	≥ 5

#### Exclusion criteria

(1) Patients with unclear preoperative injury; (2) patients transferred to intensive care unit after surgery; (3) patients who do not comply with postoperative discharge instructions.

### Data collection

1. Basic information of the patient, including gender, age, height, weight, body mass index (BMI), body type, professional characteristics, altitude of long-term residence after treatment, and complications;
2. Injury information, including the time of injury, main spinal segments of injury, cause of injury, preoperative Tlics score, preoperative Frankel grading, and VAS score at rest;
3. Surgical information, including how long it took to start the operation after the injury (days), the duration of the operation (h), and bleeding volume during operation.
4. The use of analgesics in the perioperative period.
5. One year follow-up after recovery, including postoperative VAS score when resting, how long can walk (day), each time walking distance (m), ODI score (the scale including pain intensity, self-reliance, extract, walking, sitting, standing, interfere with sleep, sex, social life, and travel) of 10 aspects, such as ODI index and part of the JOA (lumbocrural pain, gait, and bladder function).

6) The patients’ postoperative recovery was continuously tracked through long-term telephone follow-up.

### Statistical methods

According to postoperative ODI-pain score, the patients were divided into two groups: no pain 1 year after surgery and persistent chronic pain 1 year after surgery. Bivariate regression analysis was conducted to understand the risk factors for chronic pain prior to the variance analysis. And then, the patients were divided into three groups according to the analgesics used during the peri-treatment period. Whereafter the above data were classified into quantitative data and counting data according to data characteristics. One-way ANOVA was used for quantitative data, Bonferroni was used for multiple comparisons of positive results, and the results were expressed as mean ±standard deviation. Counting data were analyzed using the chi-square test, and the results were expressed as percentages. Rank sum test was used for Frankel classification, JOA lumbago, gait, and urine grade data. In addition, according to the difference comparison results, the correlation analysis of ODI-pain score, peri-treatment VAS score, and ODI index was conducted.

## Results

### Risk factors for chronic pain 1 year after surgery

The results of binary regression analysis showed that heavy preoperative spinal cord injury (Frankel grade A), higher peri-treatment VAS score (either 1 day before surgery or 1 month after surgery), longer time analgesic drug use, taller height, heavier body weight, and the use of codeine (compared to celecoxib and etoricoxib) were risk factors for postoperative chronic pain (Table 2).

**Table 2:** Risk factors for chronic pain 1 year after surgery

	***p*** value	OR	95% C.I. of OR	
			Lower limit	Upper limit
Preoperative Frankel grade A (Frankel grade E#)	0.018	10.983	1.502	80.323
VAS score 1 month after surgery	0.000	5.561	3.816	8.104
Codeine (celecoxib #)	0.000	3.764	2.24	6.323
Codeine (etoricoxib #)	0.000	3.619	2.099	6.24
Changes of VAS before and after peri-treatment	0.005	3.137	1.418	6.941
VAS score on the day before surgery	0.000	2.911	1.952	4.343
Days of use of postoperative analgesics	0.000	1.849	1.446	2.364
Days of use of preoperative analgesics	0.000	1.373	1.216	1.550
Height, cm	0.026	1.026	1.003	1.050
Weight, kg	0.022	1.024	1.003	1.044
Longest walking distance 1 year after surgery, m	0.047	0.999	0.999	1.000

*Abbreviation*: *cm* = centimeter, *kg* = kilogram, *m* = meter, *OR* = odds ratio, *C.I.* = confidence interval

Note: (#): reference variable

### Basic characteristics of the patients

**Fig. 1:**
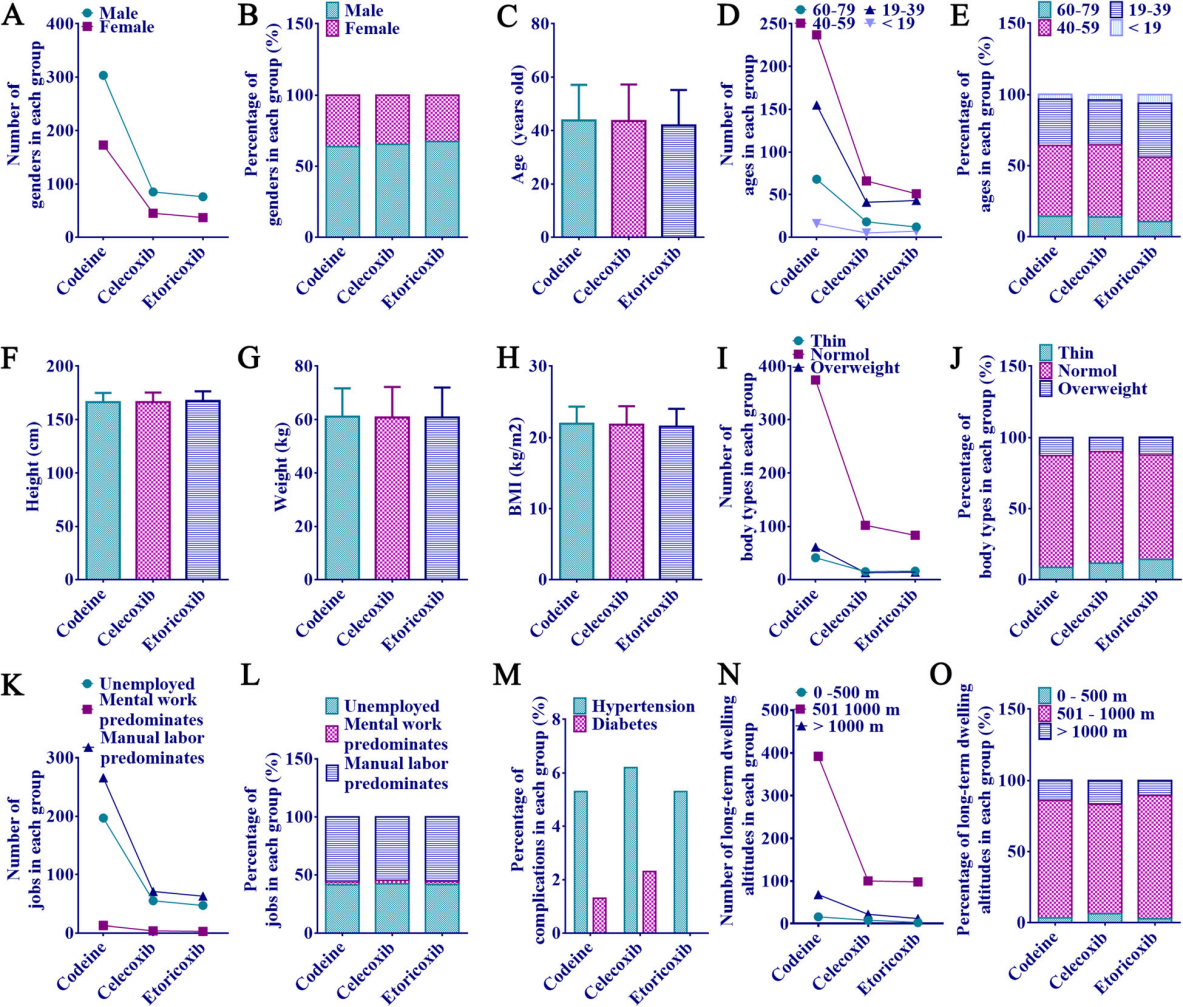
Basic characteristics of the patients. **a** Number of different genders. **b** Percentage of different genders. **c** Age value (years old). **d** Number of different age groups. **e** Percentage of different age groups. **f** Height (cm). **g** Body weight (kg). **h** BMI value (kg/m^2^). **i** Number of different body types. **j** Percentage of different body types. **k** Number of different occupations. **l** Percentage of different occupations. **m** Percentage of different comorbidities. **n** Number of groups of different altitudes of long-term residence. **o** Percentage of groups with different altitudes of long-term residence

There existed no statistical differences in gender (Fig. 1a, b), age (Fig. 1c, e), height (Fig. 1f), weight (Fig. 1g), BMI (Fig. 1h), body type (Fig. 1i, j), occupation (Fig. 1k, l), complications (Fig. 1m), and the altitude of a long-term residence (Fig. 1n, o) among the three groups (*p* > 0.05).

### Injury and condition assessment of the patients

**Fig. 2:**
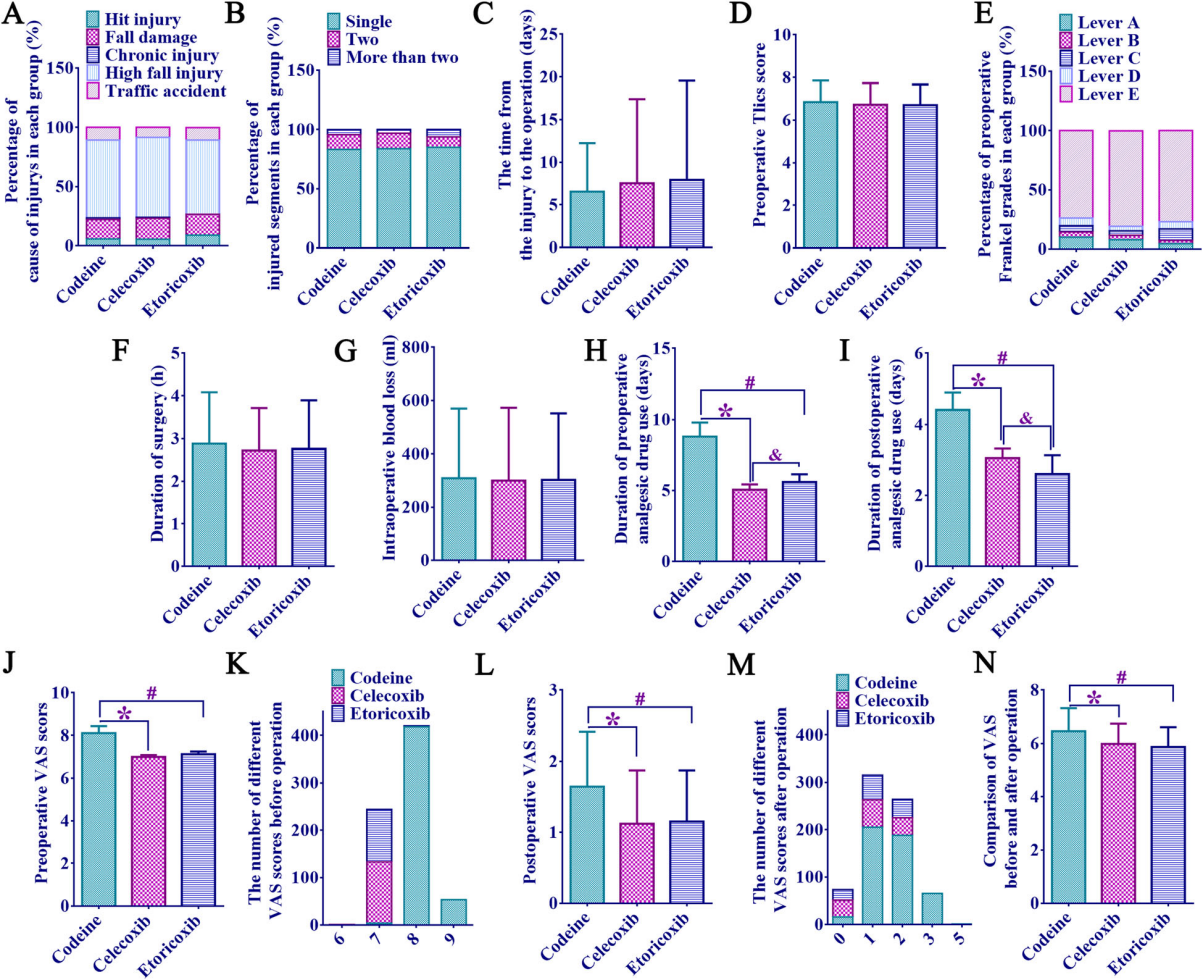
Assessment of the patient’s injury and condition, duration of perioperative analgesics, and pain score. **a** Percentage of different causes of injury. **b** Percentage of different injured segments. **c** Time from injury to operation (days). **d** Preoperative Tlics score. **e** Preoperative percentages of different Frankel grades. **f** Operation time (h). **g** Intraoperative blood loss (ml). **h** Time of preoperative analgesic drug use (days); **i** Use time of postoperative analgesics (days). **j** VAS rest assessment 1 day before surgery. **k** Number of different VAS scores before surgery. **l** VAS at resting assessment 1 month after surgery. **m** Number of different VAS scores after surgery. **n** Comparison of VAS differences before and after operation. * The difference between codeine and celecoxib groups was statistically significant, *p* < 0.01. # The difference between the codeine and etoricoxib groups was statistically significant, *p* < 0.01. & The difference between codeine and celecoxib groups was statistically significant, *p* < 0.01

There existed no statistically conspicuous differences among the three groups in the cause of injury (Fig. 2a), number of injured segments (Fig. 2b), time from injury to operation (days) (Fig. 2c), preoperative Tlics score (Fig. 2d), Frankel classification (Fig. 2e), operation time (h) (Fig. 2f), and intraoperative blood loss (ml) (Fig. 2g) (*p* > 0.05).

### The use and pain assessment of analgesics in the peri-therapeutic period

The number of days of use of analgesics in peri-treatment period (from diagnosis to departure from hospital) in the codeine group was significantly higher than that in the celecoxib and etoricoxib groups (preoperation 8.80 ± 0.98 vs 5.05 ± 0.37 vs 5.60 ± 0.54; postoperation 4.40 ± 0.49 vs 3.05 ± 0.27 vs 2.60 ± 0.53, *p* < 0.01, Fig. 2h, i).

VAS score of the codeine group was obviously higher than that of the other groups (8.10 ± 0.33 vs 6.99 ± 0.09, 8. 10 ± 0.33 vs 7.12 ± 0.13, *p* < 0.01) 1 day before the operation, and there existed no statistical significance between the celecoxib and etoricoxib group (*p* > 0.05, Fig. 2j). It was worth noting that the VAS scores of patients in the codeine group were all 7 points or above, mainly 8 points (418/476, 88.0%). And patients in the celecoxib and etoricoxib groups had a preoperative VAS score of 6 or 7 (Fig. 2k).

VAS scores of the codeine group at resting 1 month after surgery were higher than those of the celecoxib and etoricoxib group (1.64 ± 0.77 vs 1.12 ± 0.75, 1.64 ± 0.77 vs 1.15 ± 0.72, *p* < 0.01, Fig. 2l). Although patients in the codeine group had a higher VAS score 1 month after surgery than those in the other two groups, only one patient had a score of 5, and the rest had mild pain (VAS score ≤ 3) (Fig. 2m).

The difference of VAS scores before and after operation in the codeine group was greater than that in the celecoxib and etoricoxib group (6.45 ± 0.87 vs 5.98 ± 0.75, 6.45 ± 0.87 vs 5.87 ± 0.73, *p* < 0.01), there was no obvious difference between celecoxib and etoricoxib group (*p* > 0.05, Fig. 2n).

### Recovery after 1 year follow-up

**Fig. 3:**
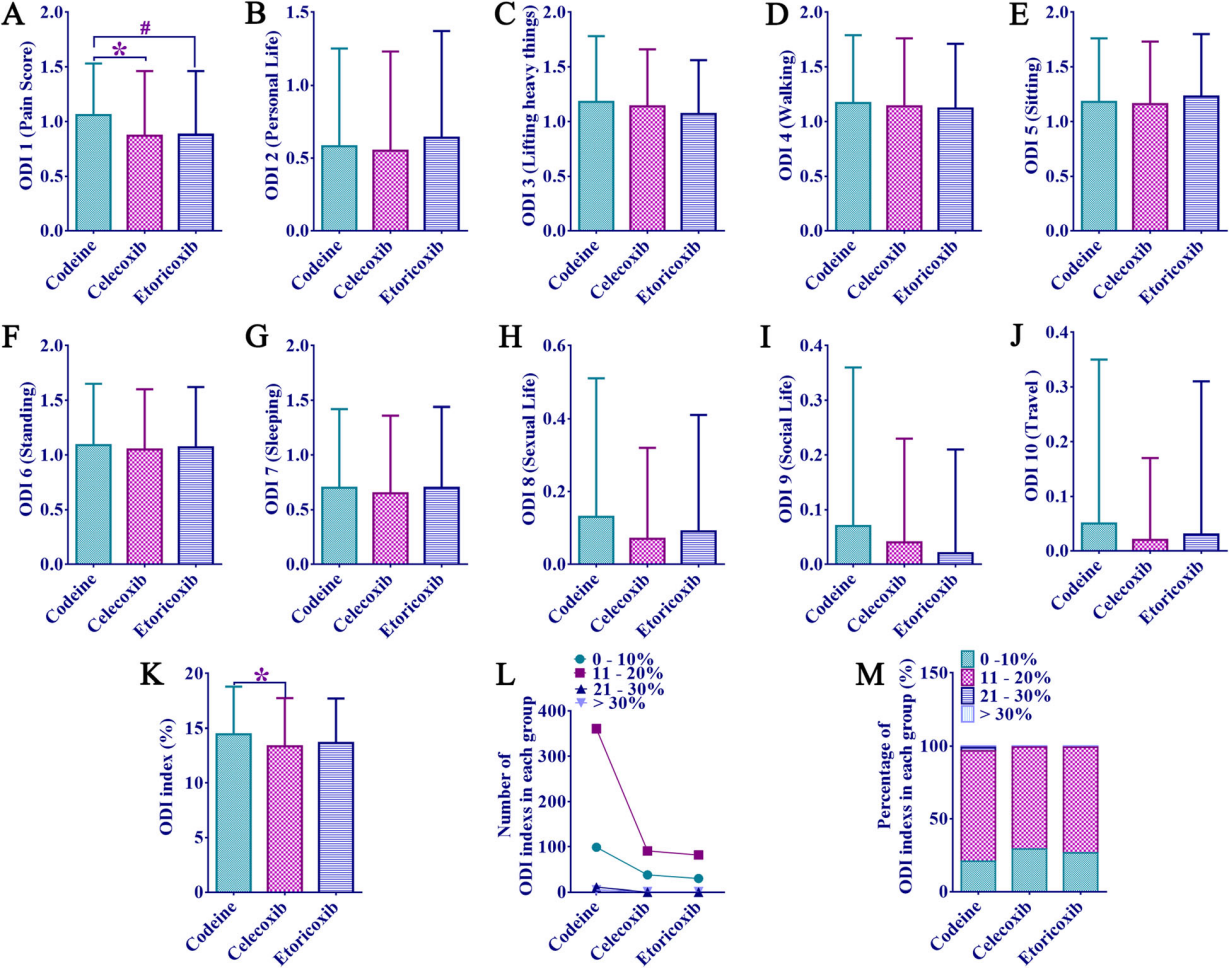
Postoperative ODI score. **a** ODI-1 (pain assessment). **b** ODI-2 (personal life). **c** ODI-3 (weight lifting). **d** ODI-4 (walking). **e** ODI-5 (seated). **f** ODI-6 (walking). **g** ODI-7 (sleeping). **h** ODI-8 (sexual life). **i** ODI-9 (social life). **j** ODI-10 (travel). **k** ODI index number. **l** The number of segments of different ODI indexes. **m** Percentage of different ODI index segments. *There were significant differences between the codeine group and the celecoxib group, *p* < 0.01. #There were significant differences between the codeine group and the etoricoxib group, *p* < 0.01

**Fig. 4:**
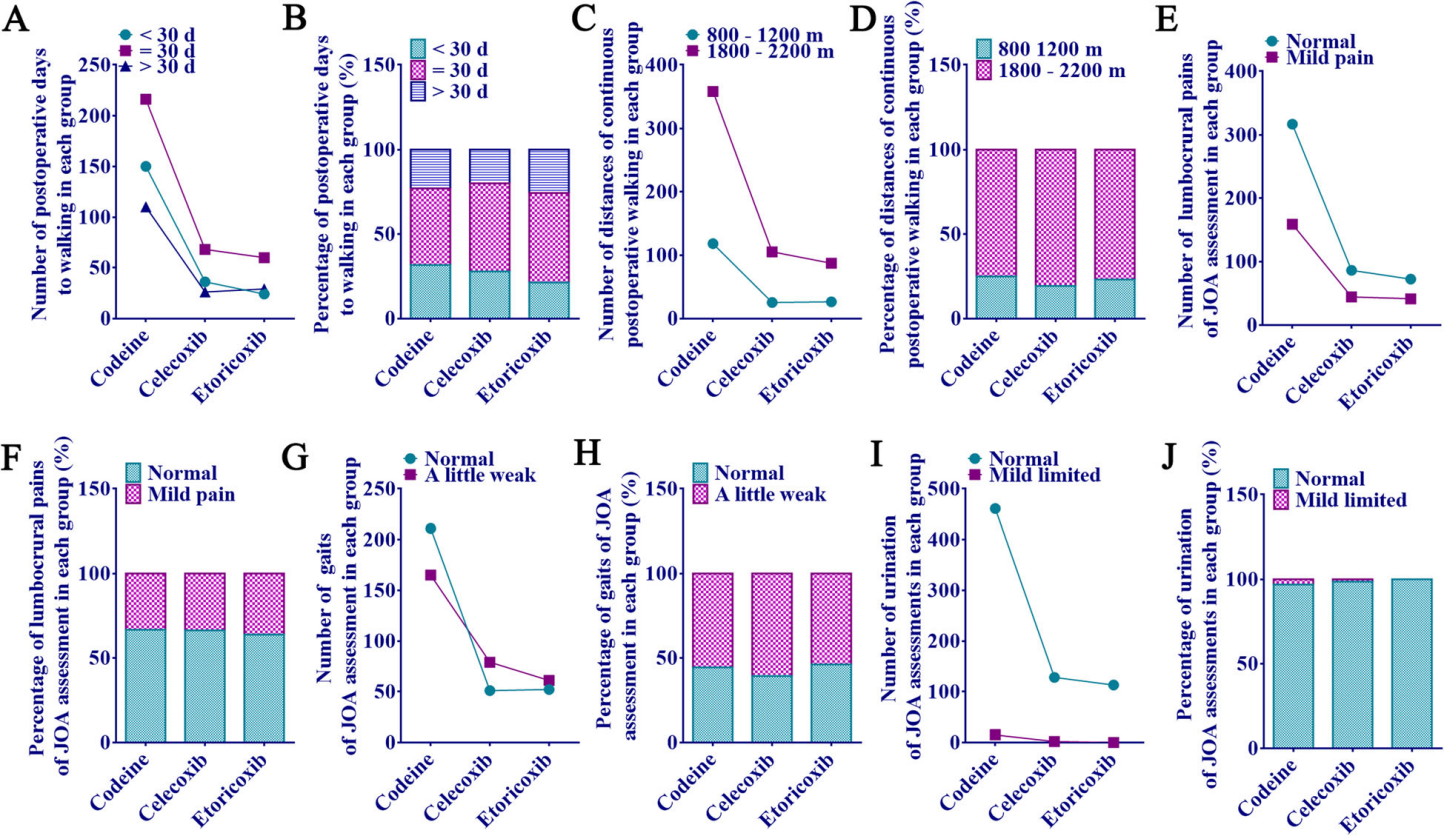
Postoperative walking condition and JOA score. **a** The number of different days from postoperative to walking. **b** Percentage of different days from postoperative to walking. **c** The number of different distances walked after surgery. **d** The percentage of different distances walked after surgery. **e** The number of different degrees of lumbar and leg pain assessed by the JOA in each subgroup. **f** The percentage of different degrees of lumbar and leg pain assessed by the JOA in each subgroup. **g** The number of different states of gait assessed by the JOA in each subgroup. **h** The percentage of different states of gait assessed by the JOA in each subgroup. **i** The number of urinary function limitations assessed by the JOA in each subgroup. **j** The percentage of urinary function limitations assessed by the JOA in each subgroup

After the same surgery, there were no statistical significance among the three groups on the postoperative ODI score (personal life, extract, walk, sit, stand, sleep, sex, social life, travel, ODI index grouping) (Fig. 3b–j and Fig. 3l, m), postoperative walking condition (Fig. 4a–d) and JOA score (lumbocrural pain, gait, and postoperative bladder function) (Fig. 4e–j) (*p* > 0.05). In terms of ODI-1 pain score, the codeine group was significantly higher than the other two groups (1.06 ± 0.47 vs 0.87 ± 0.59, 1.06 ± 0.47 vs 0.88 ± 0.58, *p* < 0.01), and the comparison between celecoxib and etoricoxib group was not significant (*p* > 0.05, Fig. 3a). In addition, the ODI index of the codeine group was higher than that of the celecoxib group (14.44 ± 4.35 vs 13.35 ± 4.39, *p* < 0.01) and the etoricoxib group (Fig. 3k). In other words, the codeine group had worse overall functional recovery 1 year after surgery than those in the other two groups.

### Correlation analysis of peri-treatment VAS score and postoperative ODI-1 pain score

The results of correlation analysis showed that ODI-pain score were correlated with VAS score on the day before surgery, VAS score on the month after surgery, peri-treatment VAS variation, and ODI index. The correlation coefficients were 0.135 (*p* < 0.01), 0.293 (*p* < 0.01), 0.085 (*p* = 0.023 < 0.05), and 0.370 (*p* < 0.01), respectively.

### Comparison of the incidence and severity of chronic pain in each group 1 year after surgery

Patients in the codeine group had a higher incidence of chronic pain after surgery than those in the celecoxib and etoricoxib groups (Table 3). In addition, the ODI-1 (pain score) of each subgroup was compared, and the results showed that the majority of chronic pain in each subgroup was mild pain, and about 11.50–13.90% of patients had moderate pain (Table 3).

**Table 3:** Comparison of the incidence and severity of chronic pain in 1 year after different analgesics were used in the peri-treatment period of thoracolumbar fracture

		Codeine	Celecoxib	Etoricoxib	***p*** value
		***N*** = 476	***N*** = 130	***N*** = 113	
**ODI-1 (pain score) 1 year after surgery**	**0 No pain**	8.00% (38/478)	24.60% (32/130)	23.90% (27/113)	< 0.001*
	**1 Mild pain**	77.90% (371/478)	63.80% (83/130)	64.60% (73/113)	
	**2 Moderate pain**	13.90% (66/478)	11.50% (15/130)	11.50% (13/113)	
	**3 Severe pain**	0.20% (1/478)	0.00% (0/130)	0.00% (0/113)	
**Chronic pain was present after surgery**		92.00% (438/478)	75.40% (98/130) #	76.10% (86/113) #	< 0.001*

*Abbreviation: N* = numbers, *ODI* = Oswestry dysfunction indexNote: *The overall difference was statistically significant, # Vs codeine group, The difference was statistically significant, *p* < 0.01

## Discussion

In this study, a total of 719 patients with thoracolumbar fracture undergoing the same surgical treatment were collected and divided into three groups according to the analgesic drugs used during the peri-treatment period. Regression analysis showed that preoperative severe spinal cord injury, more severe pain during the peri-treatment period (especially after the operation of a month’s worth of pain degree), more pronounced changes in pain, codeine use (with celecoxib or etoricoxib as reference), and longer use of analgesics were risk factors for postoperative chronic pain. And the subsequent difference analysis showed that there was no statistical significance in the basic characteristics, injuries, and surgical conditions of each subgroup. All patients received the same operative treatment and followed the same discharge instructions in this study. That is, excluding the severe effects of spinal injury, compared with celecoxib and etoricoxib, acetaminophen dihydrocodeine has a relatively longer period of used and improved pain levels more significantly 1 month after surgery, but the patients had worse overall functional recovery 1 year after surgery (higher ODI index), due to more postoperative chronic pain (higher ODI-pain). Notably, almost all patients had no or only mild pain 1 month after surgery. At the same time, the correlation analysis showed that the peri-treatment pain score was correlated with the severity of postoperative chronic pain. And in addition, the probability of postoperative chronic pain was higher in all groups (75.4–92%), especially in patients who received acetaminophen dihydrocodeine analgesia during the peri-therapeutic period.

### For patients with severe preoperative pain, it was necessary to use a combination of strong analgesics and analgesics with anti-inflammatory analgesics

As an opioid alkaloid, acetaminophen dihydrocodeine, similar to morphine, has effective in reducing the pain of nerve damage caused by fractures [11], but lacks anti-inflammatory effects [12]. As COX-2 inhibitor drugs, celecoxib and etoricoxib can inhibit pain hypersensitivity, achieve dual anti-inflammatory, and analgesic effects [13, 14]. An the comparison of VAS scores of the two drugs showed no statistical difference. In addition, a large number of studies have shown the significant role of opioids in acute analgesia [15–17], and the results of this part of the study was consistent with existing literature reports. That is, compared with celecoxib and etoricoxib, acetaminophen dihydrocodeine had better acute analgesic effect. However, while almost all patients had no or only mild back pain 1 month after surgery, acetaminophen dihydrocodeine treatment was associated with a higher incidence of chronic pain after surgery. In addition, except the peri-treatment pain score and ODI index, there were no significant differences in age, gender, height, weight, preoperative spinal cord injury degree, and postoperative JOA score among all subgroups. Pain from lumbar fractures and surgical trauma was acute, but this pain often leaded to chronic pain after surgery. Acute pain was mediated by an increase in the firing rate of pain-sensing neurons, which was enhanced by local inflammatory mediators such as prostaglandins, cytokines, and bradykinins. These mediators make the local peripheral environment sensitive to pain, and at the same time, the central nervous system may become sensitive due to long-term or intense painful tissue damage [18], both of which jointly cause the occurrence of postoperative chronic pain [19]. These explains why, despite the greater improvement in acetaminophen dihydrocodeine acute analgesia, patients still end up with a higher percentage of chronic pain 1 year after surgery, compared with non-steroidal anti-inflammatory drugs. In addition, many studies have shown that postoperative pain was detrimental to patients’ functional recovery, whether acute or chronic pain [20–22]. At the same time, considering the long-term use of opioids to bring about a variety of complex effects on the body [23], such as drug resistance and addiction, we suggested that the combination of drugs with multiple mechanisms of action was beneficial to reducing the percentage of postoperative chronic pain and improving the overall functional recovery of patients after surgery.

### A persistently high pain score during treatment of thoracolumbar fractures was a risk factor for postoperative chronic pain

In this study, acetaminophen dihydrocodeine-administered analgesia patients had higher pain scores throughout the treatment period than patients on celecoxib and etoricoxib. And regression analysis showed that more severe pain at each time point was a risk factor for postoperative chronic pain. Previous studies have shown that incomplete perioperative analgesia can cause chronic pain in patients after surgery [18, 19]. In addition, these studies have shown that prolonged mild to moderate pain can lead to peripheral and central “sensitization” of the body to pain, increasing the probability of chronic pain after surgery.

### Strengths and limitations

In this study, we found that for patients with thoracolumbar fracture, acetaminophen dihydrocodeine was more effective than celecoxib and etoricoxib in the acute analgesia of surgical trauma, but it was more likely to cause postoperative chronic pain due to lack of anti-inflammatory effect, which was not conducive to postoperative functional recovery. It was suggested that the analgesics with different mechanisms mentioned above should be used together in the treatment period of such surgery to reduce the degree of postoperative chronic pain and improve the recovery of postoperative function. Compared with the existing studies, we systematically compared the effects of analgesics with different analgesia mechanisms on postoperative acute and chronic pain, and postoperative rehabilitation in patients with thoracolumbar fracture during peri-treatment, and tracked the patients’ recovery through software for a long time. Of course, due to the complexity and particularity of clinical cases, this study also had some limitations. For example, it was impossible to judge the impact of lifestyle on this study because lifestyle habits were difficult to define and categorize. In this study, we tried our best to remind patients through self-made application and provide postoperative rehabilitation guidance so as to avoid the difference in research results caused by lifestyle. In addition, patients often had coexisting severe pain before inclusion in this study, making it difficult to measure their pain sensitivity prior to this study. This can only be avoided to a certain extent by calculating the differences in the basic information of each subgroup of patients through statistical methods. And this study is a retrospective analysis, so the results of this study can only be used as a basic understanding of the impact of analgesics on postoperative recovery, and further prospective studies and other research methods with higher evidence level are needed in the later stage.

## Conclusion

The acute analgesic effect of acetaminophen dihydrocodeine was higher in patients with severe pain of thoracolumbar fractures. However, due to its lack of anti-inflammatory effect, it has a higher probability of persistent mild to moderate pain throughout the treatment period and a higher proportion of postoperative chronic pain compared with celecoxib and etoricoxib, which reduces the quality of postoperative recovery. So, for patients with severe preoperative pain, it is necessary to use a combination of strong analgesics and analgesics with anti-inflammatory analgesics.

## Data Availability

The data are available from the corresponding author upon reasonable request.

## Abbreviations

BMI: Body mass index
VAS: Visual analog scale
ODI: Oswestry dysfunction index
JOA: Japanese Orthopedics Association score
COX: Cyclooxygenase
NSAID: Non-steroidal anti-inflammatory drugs
PD: Paracetamol and dihydrocodeine
